# Supplementary material for: Amplifying the effects of adding extra players during association football game-based scenarios
Source: PLoS One. 2022 Jun 24;17(6):e0270052. doi: 10.1371/journal.pone.0270052 (PMC9231771; doi:10.1371/journal.pone.0270052)
Supplement: Attachment — Submitted filename: Reviewer Answers.docx [file pone.0270052.s002.docx]

**Dear Editor,**

Thank you very much for your reassessment of our manuscript entitled “**Amplifying the effects of extra players during association football game-bases scenarios**”. We really would like to thank the Reviewers and to the Associate Editor for being always constructive in their feedback. We have included most of the suggestions made by the reviewer. Those changes are highlighted in blue color or track in change within the manuscript.

**Journal Requirements:**

Please review your reference list to ensure that it is complete and correct. If you have cited papers that have been retracted, please include the rationale for doing so in the manuscript text, or remove these references and replace them with relevant current references. Any changes to the reference list should be mentioned in the rebuttal letter that accompanies your revised manuscript. If you need to cite a retracted article, indicate the article’s retracted status in the References list and also include a citation and full reference for the retraction notice.

**Authors:** Dear team, some references were included following the revision #1 to support requests from the reviewers, where we attempted to improve our theoretical rationale. In the current review, no additional references were added to the text.

**Comments to the Author**

Reviewer #1: I am pleased to see the authors improved the quality of the manuscript with practical aspects in relation to technical/tactical behavior of LSG soccer training. I only have several minor comments this time.

**Authors**: We highly appreciate the reviewer insightful and helpful comments on our manuscript. We do believe that it helped us to improve the work, and consequently, improving its clarity and rationale.

**Reviewer #1**

Abstract. Please add information of studying participants. Furthermore, add statistical report in the result section. Please also clarify “small to moderate effects”.

**Authors**: Thank you. We have added authors age and experience. Also, statistical report was included, while the small to moderate effects were modified by the corresponding values of Cohen’s d effects. Again, thank you for helping us in the manuscript improvement.

**Reviewer #1**

Add references to support the statement, line 109-112.

**Authors**: We appreciate. References were accordingly added: “Based on the previous insights, previous research explored how players’ behaviour is modified by different numerical relations during SSG to aid coaches with practical and relevant information for practice design (6, 7, 9, 12, 15)”

**Reviewer #1**

Line 122. what do you mean about “higher numerical unbalances”.

**Authors**: Our intention it was to mention to differences higher than one player per team. The following information was added to the sentence: “In fact, exposing players to higher numerical unbalances (i.e., difference between teams of more than one player, such as 5vs3, 7vs4) is a common approach used by coaches to develop the players’ offensive and defensive behaviours in sectorial tasks (15).”

**Reviewer #1**

Line 175, you have 10 players for each team (please add information related to their training status). Should the authors include 3 additional players as studying participants?

**Authors**: We do understand the reviewer perspective. Despite being used 25 players (2 Goalkeepers, 20 outfield players and the 3 additional players), only the data from the 20 were used as consisted in the players presented in all data collection sessions and conditions. The following sentence was added: “In addition, while three additional players were used (i.e., to promote the numerical unbalance between teams), their data was not computed as they did not participate in all conditions (e.g., the 11vs11 did not include any additional player).”

Also, the following information was added to the participants training status: “All players were engaged in four training sessions per week (90 to 105 minutes per session) and had an official 11-a-side match during the weekend at a regional playing standard level.”.

Furthermore, it was added during the procedures the phase level of the competition: “All conditions were tested for two weeks. The first week was used for familiarization purposes, while the following week was used for the testing sessions. The experimental sessions were performed in non-consecutive days (i.e., with difference of two days between them) and developed during the middle of the in-season competitive period (season 2017/2018).

**Reviewer #1**

Line 216, .what is the resting interval between the testing days?

**Authors**: Thank you for raising this question. It were collected with a difference of 2 days.

**Reviewer #1**

Line 228, “officially sized” is redundant.

**Authors**: Thank you. It was deleted accordingly.

**Reviewer #1**

Line 237, add humidity of the testing days.

**Authors**: We appreciate this suggestion. It was accordingly added.

**Reviewer #1**

Lin2, 249, the location of filming? I suppose to be the central of the ground if only one camera.

**Authors**: Yes. It was used only one, and in this respect, it was placed in the central zone of the pitch at a height of 2m. The following information was added: “The digital video camera was fixed at a 2-m height and aligned in the midfield part of the pitch.”

**Reviewer #1**

Table 1, p value of Dis Covered while sprint should be 0.039.

**Authors**: Thank you for identifying this mistake. We have updated the table accordingly.

**Reviewer #1**

Clarify small/moderate, small to moderate …effect.

**Authors**: We do appreciate the reviewer corrections. We have modified it accordingly. To avoid possible mistakes, we have updated as in the abstract. That is, the information was modified by the corresponding Cohen’s d values.

**Reviewer #1**

X2 should be superscript. Revised in the result section.

**Authors**: Thank you. It was modified accordingly.

**Reviewer #1**

Line 478, what do you mean “as the pitch was kept constant”?

**Authors**: We appreciate for raising this point. Our intention it was to note that it would be expected to decrease the distance as more players were added to the same space. However, we decided to delete that specific part from the text to avoid misconceptions.

**Reviewer #1**

Line 523, remove “available”.

**Authors**: Thank you. It was deleted accordingly.

**Reviewer #1**

Line 500, revise “balls” to “ball”.

**Authors**: We appreciate for this suggestion. It was modified accordingly.

**Reviewer #1**

Concise the sentences, line 513-516 and conclusion.

**Authors**: We attempted to be more concise in the lines 513-516, however, we are unsure if we were modified in the proper space. Sorry if we fail to correct the proper section. In addition, also the conclusion was also modified to become shorter.

-------------------------------------------------------------------------------------------------------------------

**Reviewer #2**

I would like to congratulate the authors with the final manuscript. I believe they addressed all my comments/suggestions and I think the final version is really good. Well done!

Dear reviewer, again, we would like to say thank for your contribution and expertise that helped us to improve the current work.
